# Intermittent fasting promotes HFSC death to inhibit hair growth

**DOI:** 10.1093/lifemeta/loaf006

**Published:** 2025-02-19

**Authors:** Abigail Benvie, Valerie Horsley

**Affiliations:** Department of Molecular, Cellular, and Developmental Biology, Yale University, 219 Prospect Street, New Haven, CT 06511, United States; Department of Molecular, Cellular, and Developmental Biology, Yale University, 219 Prospect Street, New Haven, CT 06511, United States

**Figure 1:**
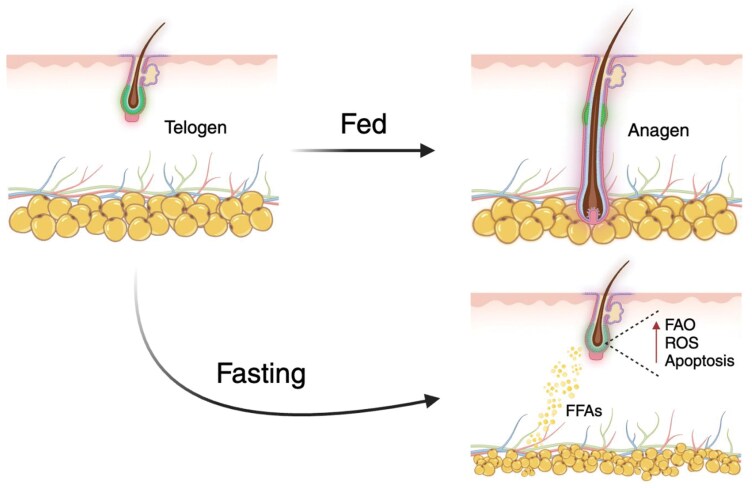
Under fed conditions, HFSCs undergo proper activation and hair follicles transition from telogen to anagen. In contrast, fasting triggers dermal adipocyte lipolysis, releasing FFAs into the surrounding niche to be utilized by HFSCs. This metabolic reprogramming induces apoptosis in HFSCs, inhibiting hair follicle regeneration.

**In the last decade, intermittent fasting (IF) has risen in popularity due to its ability to promote significant weight loss and improve overall cardiometabolic health. In a recent study published in *Cell*, Chen and colleagues found that fasting-induced lipolysis of dermal adipocytes triggers hair follicle stem cells (HFSCs) to undergo apoptosis, thus inhibiting hair follicle regeneration in both mice and humans (Fig. 1)**.

Intermittent fasting (IF) regimens have gained traction recently with various studies demonstrating that these diets offer a simple, yet highly effective method of losing weight. Put simply, IF can be described as alternating cycles of food restriction followed by consumption. The two most prominent methods include fasting by alternating days and time-restricted eating. As its name suggests, alternative day fasting involves 1 day of fasting followed by 1 day of consuming at will. Alternatively, time-restricted eating confines the time window within a single day in which one eats versus fasts—often 8 h of consumption *ad libitum* and 16 h of fasting. In addition to weight loss, numerous studies in both mice and humans report improvements in various other metabolic parameters including blood pressure, circulating levels of glucose, insulin, and lipids, inflammation, and oxidative stress [1]. While the impact of IF on these general markers of human health has been well-characterized, how fasting impacts tissue health and regeneration is not well understood.

Proper tissue homeostasis and repair rely upon specific populations of adult stem cells. Within the tissue, these stem cells reside within their specific “niches”—a highly specialized, heterogeneous microenvironment that is critical for regulating stem cell behavior and function. Niche cells and components communicate with resident stem cells, as well as with one another, to respond to fluxes in environmental cues including stress, tissue injury, and nutrient changes. However, the molecular mechanisms, by which IF may influence stem cell dynamics, and therefore tissue renewal, remain unclear.

Several reports have demonstrated the beneficial role of fasting on the adult stem cell pool in a variety of tissues. For example, IF induced a metabolic shift in intestinal stem cells in the gut towards utilizing fatty acid oxidation and increasing their proliferative capacity [2]. Furthermore, periods of caloric restriction were shown to induce the quiescence of muscle stem cells in skeletal muscle, thus enhancing their resilience to cellular stressors [3]. Similarly, prolonged periods of IF promoted stress resistance and self-renewal in stem cells of the hematopoietic system [4]. Taken together, these data highlight the therapeutic potential that fasting may hold in targeting adult stem cells. However, studies in other tissues—specifically, highly regenerative tissues, such as skin—remain lacking.

Skin is a highly regenerative organ composed of several stem cell populations that maintain the epidermal barrier and its appendages, including the hair follicle [5]. Hair follicle stem cells (HFSCs) maintain bouts of hair growth (anagen) during the hair follicle cycle which consists of stages of hair follicle recession (catagen) and rest (telogen) [5]. To address how intermediate fasting impacts HFSCs, the Zhang group examined hair growth after different feeding regimes. Despite enhanced glucose tolerance of mice with intermediate fasting compared to mice fed *ad libitum*, hair growth was impaired as hair follicles remained in telogen [6]. Thus, while IF may be metabolically beneficial, rejuvenation of stem cells within skin tissue may be impacted negatively.

Surprisingly, when Chen *et al.* [6] analyzed HFSC dynamics after the first fasting period of the diet regimen, they found no differences in HFSC activation. However, as the IF regimen proceeded, the newly activated HFSCs were depleted from the niche, becoming apoptotic. Interestingly, upon long periods of intermediate fasting, the repetitive cycles of HFSC activation followed by cell death depleted the HFSC pool, ultimately leading to hair follicle degeneration.

IF diets have been shown to reduce overall energy intake and impact an organism’s circadian rhythm [7]. Therefore, the authors interrogated the possibility that these alternative effects of fasting were having an indirect role in their observations thus far. Surprisingly, metabolic cage analysis revealed that control mice and mice undergoing intermediate fasting consumed similar amounts of calories, as fasted mice would compensate during the refeeding period. In addition, day versus night fasting displayed similar levels of HFSC activation, followed by apoptosis, suggesting that neither caloric intake nor alterations in circadian rhythm contribute to HFSC death.

IF triggers a drop in nutrient availability and activates several biological pathways including the mammalian target of rapamycin (mTOR) signaling pathway. However, several genetic mouse models revealed that the inhibition of the mTOR pathway did not impact fasting-induced apoptosis of HFSCs. Because fasting promotes lipid mobilization from adipocytes for energy, Chen *et al.* [6] then turned their attention towards the dermal adipocyte layer—critical niche structure involved in both hair follicle and skin regeneration [8]. They found that indeed, lipolysis, or release of lipid, of dermal adipocytes correlated with the fasting length and the appearance of apoptotic HFSCs and adipocyte lipolysis was halted by refeeding. Further strengthening the link between fasting-induced lipolysis and HFSC apoptosis, genetic inhibition of adipocyte lipolysis decreased HFSC death.

Because fasting stimulates adipocyte lipolysis to release free fatty acid (FFA) into the surrounding tissue to be utilized as energy by nearby cells via fatty acid oxidation (FAO), it was hypothesized that under the fasted state, HFSC undergoes a shift in metabolism, resulting in cell death. In alignment with this, genetic blockade of FAO in HFSC significantly reduced apoptosis, while administration of FFA directly into the tissue was sufficient to promote it. RNA sequencing of fasted HFSC confirmed this metabolic switch, while also revealing significant changes in genes associated with oxidative stress. Assays testing mitochondrial health, transmission electron microscopy, and histological analysis revealed that these elevations in oxidative stress correlated with increased levels of reactive oxygen species, dysfunctional mitochondria, and DNA damage in fasted HFSCs.

The authors additionally showed that activation of the adrenal glands upon fasting increased corticosterone and epinephrine levels to stimulate dermal adipocyte lipolysis and subsequent HFSC death. Both genetically and pharmacologically inhibiting the activity of either hormone, as well as surgically removing the adrenal glands, prevented dermal adipocyte lipolysis and HFSC apoptosis, thus allowing hair regrowth. Consistent with their data in mouse models, treating HFSCs from healthy human donors *in vitro* with FFA resulted in similar markers of oxidative stress, damaged mitochondria, and apoptosis. Lastly, to further demonstrate the clinical relevance of their findings, the authors conducted a clinical trial in which participants were randomized to either a normal diet or IF and had a small area of their scalp shaved. Replicating their mouse findings, individuals who underwent IF had significantly dampened hair growth, providing strong evidence that similar mechanisms surrounding HFSC dynamics exist between mice and humans. The authors acknowledge, however, that these studies were only performed for a short period of time. Therefore, the effects of IF on long-term hair growth in humans remain unclear.

While this study demonstrates the profound, yet surprising influence of IF on HFSC dynamics and function, further research is required to fully elucidate the mechanisms in which these diet regimens may impact not only the numerous other cell types within the skin but other tissues as well. Surprisingly, one study observed that prolonged caloric restriction in mice (6 months) increased hair length and expanded the HFSC pool. This suggests the activation of alternative pathways occurring when HFSCs experience acute cycles of fast then feed in comparison to long-term decreases in energy [9]. Additionally, a critical question that remains is how lipogenesis and dermal adipocyte hypertrophy under obesogenic conditions may influence HFSC dynamics and hair follicle growth. Would mice undergoing a high-fat diet display enhanced hair follicle regeneration in either the fed or fasted state? Similarly, would obese human beings show similar results? The aforementioned studies were performed only in young, lean individuals. In addition, how these new fasting regimens may impact the regenerative properties of injured skin at the mechanistic level remains unknown. Past work has demonstrated the critical role of adipocyte lipolysis during the wound healing process. Lipolysis of dermal adipocytes is crucial for proper initiation of the inflammatory stage of tissue repair and a subset of these dermal adipocytes undergo a fate switch towards a more myofibroblast-like identity to promote skin regeneration [8]. Some studies have reported that IF enhanced wound healing in both healthy and diabetic conditions, but whether these effects were related to fasting-induced lipolysis or activation of alternative pathways remains unclear [10]. Thus, investigating the mechanisms in which fasting-induced lipolysis may impact wound healing, specifically in both lean and obese conditions, will be of additional future interest.

Taken together, the work of Chen *et al.* [6] has revealed a complex communication network between hormone release from adrenal glands, dermal adipocytes, and HFSC which are activated via fasting treatments tested. Surprisingly, contrary to past work surrounding the effects of various fasting regimens on alternative tissue-resident stem cells and regeneration, the authors unveil the detrimental role that dietary changes have on hair follicle regeneration through inducing HFSC apoptosis and thereby inhibiting hair growth in both mice and humans. Thus, a comprehensive understanding of how IF impacts various organ systems will be critical for future therapeutic use.
